# Clinical Prescription-Protein-Small Molecule-Disease Strategy (CPSD), A New Strategy for Chinese Medicine Development: A Case Study in Cardiovascular Diseases

**DOI:** 10.3389/fphar.2019.01564

**Published:** 2020-01-22

**Authors:** Yong-Zhi Guo, Ying-Nan Jiang, Yi-Fang Li, Hiroshi Kurihara, Yi Dai, Rong-Rong He

**Affiliations:** ^1^ Guangdong Province Research and Development Center for Chinese Medicine in Disease Susceptibility, College of Pharmacy, Jinan University, Guangzhou, China; ^2^ International Cooperative Laboratory of Traditional Chinese Medicine Modernization and Innovative Drug Development of Chinese Ministry of Education (MOE), College of Pharmacy, Jinan University, Guangzhou, China; ^3^ Guangdong Province Key Laboratory of Pharmacodynamic Constituents of TCM and New Drugs Research, College of Pharmacy, Jinan University, Guangzhou, China

**Keywords:** CPSD, Chinese medicine, Chinese materia medica, traditional Chinese medicine, drug discovery, cardiovascular diseases

## Abstract

Chinese medicine is a national treasure that has been passed down for thousands of years in China. According to the statistics of the World Health Organization, there are currently four billion people in the world who use Chinese medicine to treat diseases, accounting for 80% of the world’s total population. However, the obscurity of its theory, its unmanageable quality, its complex compositions, and the unknown effective substances and mechanisms are great obstacles to the internationalization of Chinese medicine. Here, we propose a new strategy for the development of Chinese medicine: the clinical prescription (C)-protein (P)-small-molecule (S)-disease (D) strategy, namely the CPSD strategy. The strategy uses clinical prescriptions as the source of medicine and uses computer simulation technology to find small-molecule drugs targeting therapeutic proteins for treating specific diseases so as to deepen awareness of the value of Chinese medicine. At the same time, this article takes cardiovascular drug development as an example to introduce the application of CPSD, which will be instrumental in the further development, modernization, and internationalization of Chinese medicine.

## Introduction

Chinese medicine, also known as Chinese materia medica (CMM), is one of the major aspects of Traditional Chinese Medicine (TCM) (Xu et al., 2013). Its long history of application has benefited Chinese, Asians, and so on (Han et al., 2017). Although it is gradually receiving more attention, CMM can only be partially approved as complementary or alternative medicine due to a lack of objective and quantitative evaluation criteria (Chan et al., 2010; Hao et al., 2017). In the year 2015, Youyou Tu won the Nobel Prize for her contribution to the world-focused antimalarial drug, artemisinin, causing people to gradually realize that CMM is a huge treasure trove of drug development resources (Kong and Tan, 2015). Therefore, a proper CMM development strategy is urgently required.

**Figure 1:**
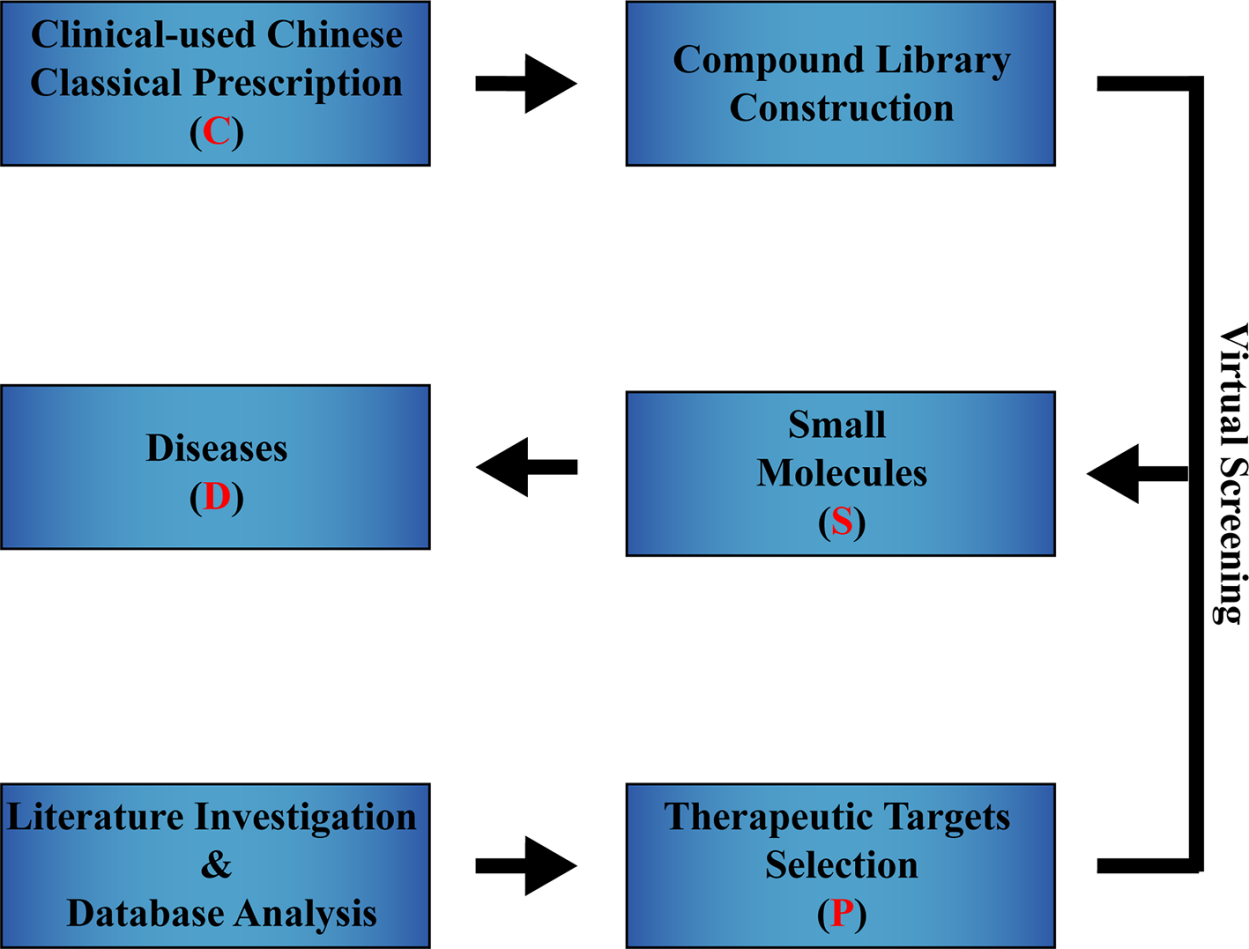
Overall process of the CPSD strategy. Firstly, CCCPs for treating a certain disease are collected, and the reported compounds contained in these CCCPs are summarized to construct a compound library (C of CPSD). After investigating the literature and analyzing databases, therapeutic target information related to a certain disease are obtained (P of CPSD). Small-molecule regulators of the therapeutic targets, which can be acquired by virtual screening technology (S of CPSD), have the potential to be candidate drugs against a certain disease (D of CPSD).

Here, we propose the **c**linical prescription-protein-small molecule-disease strategy, abbreviated as CPSD. CPSD refers to a drug development strategy that uses clinically used Chinese classical prescriptions (CCCP) as sources of potential drugs to screen for small molecules that regulate specific targets, thereby developing drugs and treating diseases (**Figure 1**). In order to explain the CPSD strategy in more detail, we use its application in the development of cardiovascular disease drugs as an example to demonstrate its value in the development of CMM.

## C: CCCP Against Cardiovascular Diseases

“ShangHanLun” (“Treatise on Febrile Diseases”), a masterpiece published during the Eastern Han dynasty of China, covered a variety of Chinese classical prescriptions against diseases (Han et al., 2017). According to TCM theory and modern medical experience, Chinese medical workers have made appropriate adjustments and deletions to the classical Chinese prescriptions to form CCCP for the treatment of diseases such as cardiovascular diseases (**Supplementary Table 1**).

### CCCP Against Myocardial Ischemia

Danlou Decoction (DL), consisting of 10 CMMs such as *Salvia miltiorrhiza* Bunge (Danshen), *Ligusticum chuanxiong* Hort. (Chuanxiong), *Trichosanthes kirilowii* Maxim. (Gualou), and so on, has taken advantage of TCM practice for the treatment of atherosclerotic plaques and ischemia-reperfusion injury (Mao et al., 2016). Buyang Huanwu Decoction (BHD) was indicated to be beneficial for cerebrovascular diseases like myocardial ischemia in both clinical trials and animal model experiments (Chen et al., 2017a). Sheng-Mai-San (SMS) is composed of *Panax ginseng* C.A.Mey. (Renshen), *Ophiopogon japonicus* (Thunb.) Ker Gawl. (Maidong), and *Schisandra chinensis* (Turcz.) Baill. (Wuweizi) and has demonstrated clinical efficacy in the treatment of ischemic heart disease in China (Yang et al., 2017b).

### CCCP Against Myocardial Infarction

Danhong Decoction (DH) is chiefly composed of two herbs, *Salvia miltiorrhiza* Bunge (Danshen) and *Carthamus tinctorius* L. (Honghua). It is widely used for the treatment of various vascular diseases including occlusive vasculitis, coronary disease, and myocardial infarction in China (Qian et al., 2018a). Chaihu Longgu Mulitang (BFG), a Chinese classical prescription (CCP) that promotes blood circulation to remove blood stasis, protecting cardiomyocytes and inhibiting platelet aggregation, is applied against acute myocardial infarction (Wang et al., 2018a).

### CCCP Against Coronary Heart Disease

Yiqi Fumai Decoction (YQFM) has been declared to significantly improve myocardial contraction and diastolic function and the pumping function of the heart (Sun et al., 2012). Gualou Xiebai Baijiu decoction (GLXB), consisting of *Trichosanthes kirilowii* Maxim. (Gualou), *Atractylodes macrocephala* Koidz. (Xiebai), and liquor, was utilized to treat “chest obstruction and heart pains” in ancient China. Now, GLXB is still used clinically against coronary heart disease and angina pectoris (Lin et al., 2018). Liuwei Dihuang (LWDH), a CCP used to treat numerous diseases with symptoms of ‘Kidney-Yin’ deficiency syndrome for over 1000 years in China, has other functions such as in the treatment of coronary heart disease (Jing et al., 2017). Gegen Decoction (GG) is one of the representative CCPs for treating exogenous diseases. Modern pharmacological research showed that GG has an anti-thrombosis effect, and it is commonly used in various clinical disease fields (Li et al., 2017).

### CCCP Against Arrhythmia

Guizhi Gancao Decoction (GZGC) consists of *Cinnamomum cassia* (L.) J.Presl (Guizhi) and *Glycyrrhiza uralensis* Fisch. ex DC. (Zhigancao), a drug pair with a positive inotropic effect. GZGC can be utilized to treat diseases such as pulmonary heart disease, coronary heart disease, and cardiovascular neurosis (Ma and Lu, 2011). Clinical studies have shown that Huanglian Ejiao Decoction (HLEJ) has a significant impact on patients with atrial fibrillation or arrhythmias after viral myocarditis, with reduced incidence of adverse reactions (Fei, 2011).

### CCCP Against Hypertension

It is pointed out that in addition to its antihypertensive effect, Tianma Goutengyin (TMGTY), the CCCP for therapy against hypertension, can also reduce the risk of cardiovascular disease by improving lipid metabolism and protecting target organs (Jiao and Liu, 2017). Wuzhuyu Decoction (WZY) is a CCP with *Tetradium ruticarpum* (A.Juss.) T.G.Hartley (Wuzhuyu), *Zingiber officinale* Roscoe (Shengjiang), *Panax ginseng* C.A.Mey. (Renshen), and *Ziziphus jujuba* Mill. (Dazao) that is used clinically to treat hypertension and heart failure (Du, 1999).

## P: Proteins or Pathways Associated With Cardiovascular Diseases

**Figure 2:**
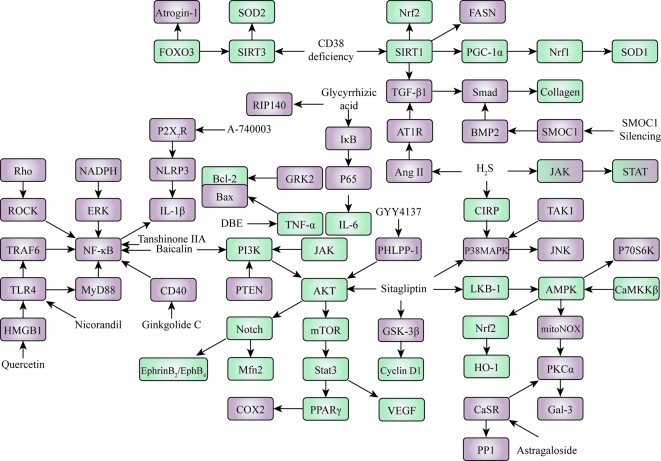
Proteins or pathways associated with cardiovascular diseases. Green and red indicate that activating/upregulating or inhibiting/downregulating these proteins was reported to be beneficial for treatments of cardiovascular diseases, respectively. A mixture of red and green means that both upregulation and downregulation of these proteins were reported to attenuate cardiovascular diseases.

Regulation of a variety of proteins is closely linked to cardiovascular development and cardiovascular disease treatment. These proteins can serve as drug targets for cardiovascular disease drug design and development. For the criteria of selection of proteins or targets related to cardiovascular diseases, we investigated all of the literature related to cardiovascular disease pathways published between 2001 and 2018. Through further screening, we removed repeated reports and extracted proteins or targets where there is clear evidence (such as *in vitro* and *in vivo* experiments) that they are associated with cardiovascular disease, and these are reflected in the present article. In addition, we constructed a protein network based on the upstream-downstream relationship reported in the articles, which may help to systematically understand the overall process of cardiovascular disease development and facilitate the progress of anti-cardiovascular drugs (**Figure 2**).

### PI3K-AKT and Related Pathways

The Phosphatidylinositol-3 kinase (PI3K)-protein kinase B (AKT)-mammalian target of the rapamycin protein (mTOR) signaling pathway can be activated by Dragon’s Blood extract (DBE), subsequently regulating downstream targets like vascular endothelial growth factor (VEGF), cyclooxygenase 2 (COX2), and peroxisome proliferator-activated receptor gamma (PPARγ) in cardiac tissue, to exert cardio-protection against myocardial injury (Li et al., 2018a). PI3K/Akt can also be activated by baicalin, a CMM component that can suppress nuclear factor-kappa B (NF-κB) signaling to inhibit myocardial apoptosis and inflammation (Luan et al., 2019). The cluster of differentiation 40 (CD40)/NF-κB signaling pathway and downstream inflammatory cytokine expression is suppressed by ginkgolide C to alleviate myocardial ischemia/reperfusion (MI/R)-induced inflammatory injury (Zhang et al., 2018b). The toll-like receptor 4 (TLR4)/myeloid differentiation primary response protein 88 (MyD88)/NF-κB pathway can be inhibited by nicorandil to prevent coronary microembolization-induced myocardial injury (Su et al., 2018). Inhibition of the high-mobility group box-1 (HMGB1)/TLR4/TRAF6/NF-κB signaling pathway is beneficial for treatments of MI/R injury and acute myocardial infarction (AMI) (Sun et al., 2015; Dong et al., 2018). Besides, the Rho/Rho-associated coiled-coil forming protein kinase (ROCK)/NF-κB signaling pathway can be inhibited by crocetin ester to exert a cardioprotective effect against AMI (Huang et al., 2016). By targeting the nicotinamide adenine dinucleotide phosphate (NADPH)/extracellular regulated protein kinase (ERK)/NF-κB pathway, erythropoietin can inhibit rat cardiac fibroblast proliferation, transformation, and collagen deposition (Wang et al., 2016). Receptor-interacting protein (RIP140), as well as inhibitor of NF-κB (IκB)/NF-κB p65 (p65)/IL-6, can be regulated by glycyrrhizic acid to preserve heart function by increasing cardiac antioxidants and reducing cardiomyocytes apoptosis (Yang et al., 2017a).

The activation of cold-inducible the RNA-binding protein (CIRP)/mitogen-activated protein kinase (MAPK) signaling pathway, down-regulation of the janus kinase (JAK)/signal transducer and activator of transcription (STAT) signaling pathway, and down-regulation of the angiotensin II (Ang II)/Ang II type 1 receptor (AT1R) pathway can all be induced by a simple compound, H2S, to exert cardio-protective effects like ameliorating myocardial injury, attenuating myocardial fibrosis, or improving myocardial remodeling (Liu et al., 2017; Liu et al., 2018; Zhao et al., 2018). Interestingly, in addition to its down-regulation, activating or up-regulating the JAK/STAT pathway was reported to prevent the oxidative stress-induced apoptosis of myocardial cells and inhibit myocardial fibrosis (Qu et al., 2017; Huang et al., 2018).

### AMPK/FOXO and Related Pathways

The activation of the silent information regulator 3 (SIRT3)/superoxide dismutase 2 (SOD2)/FOXO3 (Forkhead box O3)/Atrogin-1 pathway and inhibition of fatty acid synthase (FASN) can lead to the protection of the heart from oxidative stress as well as from the development of human heart failure and left ventricular dysfunction (Galasso et al., 2010; Wang et al., 2018c). The SIRT1/nuclear factor-erythroid 2-related factor 2 (Nrf2) signaling pathway is activated by honokiol to ameliorate MI/R injury (Zhang et al., 2018a). SIRT1 along with downstream TGF-β1/Smad2/3/Collagen pathways are highly involved in the prevention of fibrosis through the cardioprotective effects of caffeic acid o-nitro phenethyl ester (CAPE-oNO2). CAPE-oNO2 can also suppress oxidative stress by regulating the peroxisomal proliferators-activated receptor γ-coactivator-1α (PGC-1α)/Nrf1/SOD1 pathway to protect heart function. This protective effect is related to other pathways like Bcl-2 associated X Protein (Bax)/B-cell lymphoma-2 (Bcl-2) (Li et al., 2018b). Sitagliptin was reported to inhibit the liver kinase B-1 (LKB-1)/5′-AMP-activated protein kinas (AMPK)/Akt pathway, activate the glycogen synthase kinase-3β (GSK3-β) and p38 MAPK pathways, thereby preventing apoptosis and cardiomyopathy (Al-Damry et al., 2018). Inactivation of the TGFβ-activated kinase 1(TAK1)-p38 MAPK/c-Jun N-terminal kinase (JNK) signaling pathway protects the heart from cardiac hypertrophy and dysfunction (Li et al., 2016). The PHLPP-1/Akt/Nrf2 signaling pathway is activated by a hydrogen sulfide donor GYY4137 to protect against MI/R injury (Qiu et al., 2018). The AMPK/Nrf2/heme oxygenase-1 (HO-1) pathway, an anti-oxidative defense system, was reported to be related to the development of diabetic cardiomyopathy and the restoration of cardiac function (Li et al., 2018c). Ca2+/calmodulin-dependent protein kinase kinase β (CaMKKβ)/AMPK can modulate vagal nerve stimulation, functioning against ISO-induced myocardial ischaemia (Xue et al., 2017).

### Other Pathways

Interleukin-1β (IL-1β) can be modulated by P2X7 receptor (P2X7R) inhibition, induced by its antagonist A-740003, *via* the P2X7/NLRP3 pathway to attenuate sympathetic nerve sprouting after MI (Yin et al., 2017). SPARC-related modular calcium binding 1 (SMOC1) silencing can suppress myocardial fibrosis of mouse myocardial fibroblasts *via* regulation of the bone morphogenetic protein 2 (BMP2)/Smad signaling pathway (Wang et al., 2018d). The activation of the notch1/mitochondrial fusion-associated protein 2 (Mfn2) signaling pathway along with downstream EphrinB2/EphB4 can be activated by melatonin, preventing adverse myocardial infarction remodeling and ischemic cardiovascular diseases (Pei et al., 2016; Yang et al., 2016). The regulation of the calcium-sensing receptor (CaSR)/protein kinase C-α (PKC-α)/protein phosphatase-1 (PP-1) signaling pathway was reported to be involved in increasing cardiac diastolic function (Ji et al., 2018). Inhibition of the mitochondrial NADPH oxidase 4 (mitoNox)/PKC-α/galectin-3 (Gal-3) pathway can decrease left ventricular fibrosis following myocardial infarction (Asensio-Lopez et al., 2018).

In addition to the above, some targets or signaling pathways are closely related to the treatment of cardiovascular diseases, such as p70S6K, Cyclin D1, phosphatase and tensin homolog deleted on chromosome ten (PTEN), and G Protein-Coupled Receptor Kinase 2 (GRK2) (Glass and Singla, 2011; Lian et al., 2012; Fan et al., 2013; Chen et al., 2017b).

## S: CMM-Derived Small-Molecule Compounds/Drugs Against Cardiovascular Diseases

Hitherto, a number of small molecules isolated from CMM have been identified to function against cardiovascular diseases. Targets or pathways regulated by these small molecules, as well as experimental models used in the research process, have been partly reported (**Supplementary Table 2**). Cycloastragenol, a natural compound with various pharmacological functions derived from *Astragalus mongholicus* Bunge (Huangqi), has been demonstrated to attenuate left ventricular dysfunction and cardiac remodeling in heart failure rat models (Wang et al., 2018b). Calycosin-7-O-β-D-glucoside, an *Astragalus mongholicus* Bunge (Huangqi)-derived calycosin derivative, can reduce myocardial injury in heatstroke rats (Tsai et al., 2019). Gastrodin, a monomeric component exacted from *Gastrodia elata* Blume (Tianma), can alleviate MI/R *via* multiple mechanisms related to autophagic flux and the elimination of dysfunctional mitochondria (Fu et al., 2018). Wogonin, a dihydroxyl flavonoid compound isolated from *Scutellaria baicalensis* Georgi (Huangqin), was reported to reverse the over-activation of the PI3K/Akt pathway to relieve myocardial hypertrophy (Qian et al., 2018b). Leonurine derived from *Leonurus japonicus* Houtt. (Yimucao) can induce cardiac protection effects *in vivo via* the PI3K/AKT/GSK3β signaling pathway to have an anti-apoptosis effect (Xu et al., 2018). Honokiol is a biphenolic compound isolated from *Magnolia officinalis* Rehder & E.H.Wilson (Houpu), which can protect the heart from dysfunction *in vivo* by activating the AMPK/ULK pathway and promoting autophagy (Wei et al., 2018). Apigenin-7-O-β-D-(-6′′-p-coumaroyl)-glucopyranoside, derived from *Akebia quinata* (Thunb. ex Houtt.) Decne. (Mutong), was reported to attenuate MI/R injury by activating the AMPK signaling pathway (Feng et al., 2018). Amentoflavone is a polyphenolic compound isolated from *Selaginella tamariscina* (P.Beauv.) Spring (Juanbai), which can inhibit the renin–angiotensin system (RAS) and reduce NADPH oxidase-associated oxidative stress, leading an amelioration of cardiovascular dysfunction (Qin et al., 2018). Chicoric acid, a caffeic acid derivative isolated from *Chrysanthemum × morifolium* (Ramat.) Hemsl. (Juju), can improve heart and blood in hypobaric hypoxia yak models (Wu et al., 2018). Acacetin, a *Chrysanthemum × morifolium* (Ramat.) Hemsl. (Juhua)-derived flavonoid compound, was reported to inhibit cardiac hypertrophy and fibrosis in myocardial infarction mouse models (Chang et al., 2017). The main medicinal component of *Phragmites australis* (Cav.) Trin. ex Steud. (Luhui), barbaloin, can regulate oxidative stress and inflammation response by activation of AMPK, contributing to cardioprotective functions (Zhang et al., 2017). Resveratrol, a natural phenol generated by *Reynoutria japonica* Houtt. (Huzhang), exerts multiple beneficial effects on cardiovascular diseases such as antiatherogenic, anti-inflammatory, antihypertensive, cardioprotective effects (Cho et al., 2017). Quercetin isolated from *Styphnolobium japonicum* (L.) Schott (Huaihua) was reported to ameliorate oxidative stress, inflammation, and apoptosis in the heart of adult male diabetic rats (Roslan et al., 2017). Melatonin, a compound isolated from *Lycium barbarum* L. (Gouqizi), can exert a cardioprotective effect *via* the AMPK-PGC-1α-SIRT3 signaling pathway (Yu et al., 2017). Punicalagin, a bioactive component isolated from *Punica granatum* L. (Shiliupi), was reported to exert cardioprotective effects by restricting oxidative stress and myocardial injury in a rat model of MI/R *via* activation of the AMPK signaling pathway (Ding et al., 2017). Berberine is a component extracted from *Phellodendron chinense* C.K.Schneid. (Huangbai) found to prevent ischemia-induced heart injury in heart *via* regulation of miR-29b (Zhu et al., 2017). Naringenin, a *Citrus × aurantium* L. (Zhishi)-derived flavanone, prevented myocardial cells against prematurely induced senescence, seeming to be associated with the mitochondria (Da Pozzo et al., 2017). Diacerein, an anthraquinone compound derived from *Phragmites australis* (Cav.) Trin. ex Steud. (Luhui), can improve ventricular remodeling and reduced fibrosis and might be associated with the NF-κB pathway (Torina et al., 2015). Luteolin, a falconoid compound from *Lonicera japonica* Thunb. (Jinyinhua), was reported to protect against MI/R injury through the mechanism of regulating the ROS–MAPK pathway (Yu et al., 2015). Piceatannol, a natural phenolic compound isolated from *Rheum palmatum* L. (Dahuang), exerted various pharmacological functions like prevention of hypercholesterolemia, cardiac arrhythmia, inflammation, monocyte-endothelial cell adhesion, smooth muscle cell proliferation and migration, endothelial dysfunction, and so on (Tang and Chan, 2014). Trigonelline, an active component of *Trigonella foenum-graecum* L. (Huluba), was reported to prevent oxidative stress through the down-regulation of Hsp27 and αB-crystallin, which is beneficial for protection against myocardial injury (Panda et al., 2013). Protocatechuic acid is a phenolic compound extracted from *Ainsliaea bonatii* Beauverd (Ercha), which has a protective effect against oxidative and histopathological damage in the heart tissue of rats, preventing the toxicity of 2,3,7,8-tetracholorodibenzo-p-dioxin (TCDD) (Ciftci et al., 2013). Salvianolic acid B and Tanshinone IIA, two major extracts from *Salvia miltiorrhiza* Bunge (Danshen), were demonstrated to exert cardioprotective function through multiple targets concerned with NO production (Pan et al., 2011). A natural p300-specific histone acetyl-transferase inhibitor, curcumin, is derived from *Curcuma longa* L. (Jianghuang). Curcumin treatment with angiotensin-converting enzyme inhibitors can exert favorable effects on left ventricular systolic function in myocardial infarction in rats (Sunagawa et al., 2011). Finally, cryptotanshinone, one of the active ingredients from *Salvia miltiorrhiza* Bunge (Danshen), was reported to inhibit rat MI/R injury *in vivo via* mechanisms related to pro-inflammatory cytokines (Jin et al., 2009).

### CPSD in the Development of Cardiovascular Disease Drugs

To further define the role of the CPSD strategy in drug development, the application of CPSD in the development of cardiovascular disease drugs is shown here as an example. Linggui Zhugan Decoction (LGZG), composed of *Poria cocos* (Schw.) Wolf (Fuling), *Cinnamomum cassia* (L.) J.Presl (Guizhi), *Atractylodes macrocephala* Koidz. (Baizhu), and *Glycyrrhiza uralensis* Fisch. ex DC. (Gancao), is a CCCP employed to treat arrhythmia and heart failure (Ma and Lu, 2011). A library of compounds containing the chemical constituents of the four CMMs in LGZG named the LGZG Database was constructed with the help of the Chemistry Database (Shanghai Institute of Organic Chemistry of CAS. Chemistry Database [DB/OL]. http://www.organchem.csdb.cn. [1978-2018]). The three-dimensional geometric coordinates of the X-ray crystal structures of HO-1 (PDB code: 1UBB) and ERK (PDB code: 6DCG), two targets reported to be associated with arrhythmia, were obtained from the Protein Data Bank (PDB) (Lee et al., 2014; Yao et al., 2019). Hits were then screened from the LGZG Library using Accelrys Discovery Studio (version 3.0; Accelrys, San Diego, CA, USA) using the CDOCKER protocol. The semi-flexible docking results of CDOCKER revealed that pachymic acid and vicenin-2, as examples, formed interactions with HO-1 and ERK, respectively (**Supplementary Figure 1**). These results for compound–protein interactions between pachymic acid-HO-1 and vicenin-2-ERK were confirmed and validated against published articles (Lu et al., 2010; Kang et al., 2015). In summary, natural small molecules for specific pharmacodynamic targets against cardiovascular diseases could be screened out conveniently, quickly, and successfully by using the CPSD strategy.

## Conclusion

CMM has been used for thousands of years in China and has enormous potential for drug development. However, the diverse ingredients of CMM and the complex medical theory of TCM have meant that CMM is still not widely accepted by people all over the world.

The CPSD strategy is a CMM development strategy that integrates multidisciplinary technology. CPSD intends to use CCCP as the source of compounds and screens out small molecules for specific targets by using computer simulation technology. Using the CPSD strategy may greatly improve the successful development of small-molecule drugs with specific targets and clear indications, reducing waste of time and money. In TCM theory, CMM is believed to be the smallest drug unit, emphasizing the integrity of CMM. However, western medical scientists regard the pharmacodynamic molecule as the smallest drug unit, creating a barrier between Chinese and Western medicine. The CPSD strategy may break through the strict limits of CMM medicine and western medicine, which regards CMM in CCCP as the source of molecular drugs instead of an indivisible drug.

Since the CPSD strategy was first proposed in this paper, it should be mentioned that there is no report on the successful development of new drugs using the CPSD strategy. However, there is no doubt that this article will give researchers a new idea for the further development of Chinese medicine, and future research results achieved by using this strategy will be the best proof of the effectiveness and superiority of the CPSD strategy.

## Data Availability Statement

The datasets generated for this study can be found in the Shanghai Institute of Organic Chemistry database of traditional Chinese Medicine (http://www.organchem.csdb.cn/scdb/main/tcm_introduce.asp).

## Author Contributions

R-RH and Y-ZG conceived the concept of this perspective. Y-ZG performed the literature research and wrote the manuscript. YJ designed and made all figures and tables. HK, Y-FL, YD, and R-RH gave comprehensive advice and critically revised the manuscript. All authors approved the final version of this manuscript.

## Conflict of Interest

The authors declare that the research was conducted in the absence of any commercial or financial relationships that could be construed as a potential conflict of interest.

## Supplementary Material

Supplementary Table 1 CCCP against cardiovascular diseases.

Supplementary Table 2 CMM-derived small molecule compounds/drugs against cardiovascular diseases.

Supplementary Figure 1 Molecular docking results of pachymic acid/HO-1 and vicenin-2/ERK.
