# Supplementary material for: Clinical Prescription-Protein-Small Molecule-Disease Strategy (CPSD), A New Strategy for Chinese Medicine Development: A Case Study in Cardiovascular Diseases
Source: Front Pharmacol. 2020 Jan 22;10:1564. doi: 10.3389/fphar.2019.01564 (PMC6987446; doi:10.3389/fphar.2019.01564)

frontiers

Supplementary Material

Supplementary Tables

Supplementary Table 1 CCCP against cardiovascular diseases

Name	Constitution*	Indication	Reference
Danlou Decoction (DL)	*Salvia miltiorrhiza* Bge. (Danshen)<br>*Ligusticum chuanxiong* Hort. (Chuanxiong)<br>*Trichosanthes kirilowii* Maxim. (Gualou)<br>*Allium macrostemon* Bge. (Xiebai)<br>*Paeonia lactiflora* Pall. (Chishao)<br>*Pueraria lobata* (Willd.) Ohwi (Gegen)<br>*Alisma orientale* (Sam.) Juzep. (Zexie)<br>*Curcuma wenyujin* Y.H. Chen et C. Ling (Yujin)<br>*Drynaria fortunei* (Kunze) J. Sm. (Gusuibu)<br>*Astragalus mongholicus* Bunge (Huangqi)	Atherosclerotic plaques<br>Ischemia reperfusion injury	Mao et al., 2016
Danhong Decoction (DH)	*Salvia miltiorrhiza* Bge. (Danshen)<br>*Carthamus tinctorius* L. (Honghua)	Occlusive vasculitis<br>Coronary disease	Qian et al., 2018

		Cerebral infarction	
Yiqi Fumai Decoction (YQFM)	*Panax ginseng* C. A. Mey. (Hongshen)<br>*Liriope muscari* (Decne.) Bail (Maidong)<br>*Schisandra chinensis* (Turcz.) Baill. (Wuweizi)	Coronary heart disease	Sun et al., 2018
Gualou Xiebai Baijiu decoction (GLXB)	*Trichosanthes kirilowii* Maxim. (Gualou)<br>*Allium macrostemon* Bge. (Xiebai)<br>Liquor	Coronary heart disease	Lin et al., 2018
Chaihu Longgu Mulitang (BFG)	*Bupleurum chinense* (Chaihu)<br>Longgu (Longgu)<br>*Ostrea gigas* Thunberg (Muli)<br>*Scutellaria baicalensis* Georgi (Huangqin)<br>*Cinnamomum cassia* Presl (Guizhi)<br>Qiandan (Qiandan)<br>*Pinellia ternata* (Thunb.) (Banxia)<br>*Zingiber officinale* Rosc. (Shengjiang)<br>*Rheum palmatum* L. (Dahuang)<br>*Poria cocos* (Schw.) Wolf (Fuling)	Acute myocardial infarction	Wang et al., 2018

	Panax ginseng C. A. Mey. (Renshen)<br>*Lycium barbarum* L. (Gouqizi)		
Buyang Huanwu Decoction (BHD)	*Astragalus mongholicus* Bunge (Huangqi)<br>*Angelica sinensis* (Oliv.) Diels (Danggui)<br>*Paeonia lactiflora* PalL. (Chishao)<br>*Ligusticum chuanxiong* Hort. (Chuanxiong)<br>*Prunus persica* (L.) Batsch (Taoren)<br>*Carthamus tinctorius* L. (Honghua)<br>*Pheretima aspergillum* (E. Perrier) (Dilong)	Myocardial ischemia	Chen et al., 2017
Liuwei Dihuang (LWDH)	*Rehmannia glutinosa* Libosch. (Dihuang)<br>*Cornus officinalis* Sieb. Et Zucc (Jiuyurou)<br>*Dioscorea opposite* Thunb. (Shanyao)<br>*Alisma orientale* (Sam.) Juzep. (Zexie)<br>*Paeonia suffruticosa* Andr. (Mudanpi)<br>*Poria cocos* (Schw.) Wolf (Fuling)	Atherosclerosis	Jing et al., 2017
Sheng-Mai-San (SMS)	*Panax ginseng* C. A. Mey. (Renshen)<br>*Liriope muscari* (Decne.) Bail (Maidong)<br>*Schisandra chinensis* (Turcz.) Baill. (Wuweizi)	Ischemic heart disease	Yang et al., 2017

Guizhi Gancao Decoction (GZGC)	*Cinnamomum cassia* Presl (Guizhi)<br>*Glycyrrhiza uralensis* Fisch. (Zhigancao)	Coronary heart disease<br>Arrhythmia<br>Cardiovascular neurosis	Ma et al., 2011
Linggui Zhugan Decoction (LGZG)	*Poria cocos* (Schw.) Wolf (Fuling),<br>*Cinnamomum cassia* Presl (Guizhi)<br>*Atractylodes macrocephala* Koidz.(Baizhu)<br>*Glycyrrhiza uralensis* Fisch. (Gancao)	Coronary heart disease<br>Arrhythmia<br>Heart failure	Ma et al., 2011
Linggui Ganzao Decoction (LGGZ)	*Cinnamomum cassia* Presl (Guizhi)<br>*Glycyrrhiza uralensis* Fisch. (Zhigancao)<br>*Ziziphus jujuba* Mill. (Dazao)<br>*Poria cocos* (Schw.) Wolf (Fuling)	Cardiovascular neurosis<br>Heart failure	Ma et al., 2011
Zhigancao Decoction (ZGC)	*Glycyrrhiza uralensis* Fisch. (Zhigancao)<br>*Panax ginseng* C. A. Mey. (Renshen)<br>*Ziziphus jujuba* Mill. (Dazao)<br>*Rehmannia glutinosa* Libosch. (Dihuang)<br>*Liriope muscari* (Decne.) Bail (Maidong)<br>*Equus asinus* L. (Ejiao)	Coronary heart disease<br>Angina pectoris<br>Hypotension	Ma et al., 2011

	Cannabis sativa L. (Huomaren)<br>*Cinnamomum cassia* Presl (Guizhi)<br>*Zingiber officinale* Rosc. (Shengjiang)		
Wulingsan (WLS)	*Polyporus umbellatus* (Pers.) Fries (Zhuling)<br>*Poria cocos* (Schw.) Wolf (Fuling)<br>*Alisma orientale* (Sam.) Juzep. (Zexie)<br>*Atractylodes macrocephala* Koidz. (Baizhu)<br>*Cinnamomum cassia* Presl (Guizhi)	Chronic congestive heart failure<br>Pulmonary heart disease<br>Heart failure<br>Pericardial effusion	Ma et al., 2011
Mahuang Xixin Fuzi Decoction (MHXXFZ)	*Ephedra sinica* Stapf (Mahuang)<br>*Aconitum carmichaelii* Debx. (Fuzi)<br>*Asarum sieboldii* Miq. (Xixin)	Coronary heart disease<br>Pulmonary heart disease<br>Cardiac insufficiency	Ma et al., 2011
Xiaoxianxiong Decoction (XXX)	*Coptis chinensis* Franch. (Huanglian)<br>*Trichosanthes kirilowii* Maxim. (Gualou)<br>*Pinellia ternata* (Thunb.) (Banxia)	Coronary heart disease<br>Angina pectoris<br>Hypertensive heart disease	Ma et al., 2011
Zhishi Xiebai Guizhi Decoction (ZSXBGZ)	*Citrus aurantium* L. (Zhishi)<br>*Allium macrostemon* Bge. (Xiebai)<br>*Cinnamomum cassia* Presl (Guizhi)	Coronary heart disease<br>Angina pectoris<br>Sick sinus syndrome	Ma et al., 2011

	Magnolia officinalis Rehd. Et Wils. (Houpu)<br>*Trichosanthes kirilowii* Maxim. (Gualou)		
Renshen Decoction (RS)	*Panax ginseng* C. A. Mey. (Renshen)<br>*Zingiber officinale* Rosc. (Ganjiang)<br>*Atractylodes macrocephala* Koidz. (Baizhu)<br>*Glycyrrhiza uralensis* Fisch. (Gancao)	Coronary heart disease<br>Angina pectoris<br>Rheumatic heart disease	Ma et al., 2011
Sini Decoction (SN)	*Aconitum carmichaelii* Debx. (Fuzi)<br>*Glycyrrhiza uralensis* Fisch. (Zhigancao)<br>*Zingiber officinale* Rosc. (Ganjiang)	Coronary heart disease	Ma et al., 2011
Zhenwu Decoction (ZW)	*Aconitum carmichaelii* Debx. (Fuzi)<br>*Poria cocos* (Schw.) Wolf (Fuling)<br>*Atractylodes macrocephala* Koidz. (Baizhu)<br>*Zingiber officinale* Rosc. (Shengjiang)<br>*Paeonia lactiflora* Pall. (Baishao)	Chronic congestive heart failure<br>Dilated cardiomyopathy<br>Pulmonary heart disease	Ma et al., 2011
Mufangji Decoction (MFJ)	*Stephania tetrandra* S. Moore (Fangji)<br>Shigao (Shigao)<br>*Cinnamomum cassia* Presl (Guizhi)	Pulmonary heart disease<br>Valvular heart disease<br>Exudative pericarditis	Ma et al., 2011

	Panax ginseng C. A. Mey. (Renshen)		
Fangji Huangqi Decoction (FJHQ)	*Stephania tetrandra* S. Moore (Fangji)<br>*Glycyrrhiza uralensis* Fisch. (Gancao)<br>*Atractylodes macrocephala* Koidz. (Baizhu)<br>*Astragalus mongholicus* Bunge (Huangqi)<br>*Zingiber officinale* Rosc. (Shengjiang)<br>*Ziziphus jujuba* Mill. (Dazao)	Chronic rheumatic heart disease<br>Pulmonary heart disease<br>Coronary heart disease	Ma et al., 2011
Gegen Decoction (GG)	*Pueraria lobata* (Willd.) Ohwi (Gegen)<br>*Ephedra sinica* Stapf (Mahuang)<br>*Cinnamomum cassia* Presl (Guizhi)<br>*Zingiber officinale* Rosc. (Shengjiang)<br>*Glycyrrhiza uralensis* Fisch. (Gancao)<br>*Paeonia lactiflora* Pall. (Chishao)<br>*Ziziphus jujuba* Mill. (Dazao)	Thrombus	Li et al., 2017
Huanglian Ejiao Decoction (HLEJ)	*Coptis chinensis* Franch. (Huanglian)<br>*Equus asinus* L. (Ejiao)<br>*Scutellaria baicalensis* Georgi (Huangqin)<br>*Paeonia lactiflora* Pall. (Chishao)	Arrhythmia	Fei et al., 2011

	Jizihuang (Jizihuang)		
Danggui Shengjiang Yangrou Decoction (DGSJYR)	*Angelica sinensis* (Oliv.) Diels (Danggui)<br>*Zingiber officinale* Rosc. (Shengjiang)<br>Lamb	Arrhythmia	Fei et al., 2011
Guizhigancao Longgumuli Decoction (GZGCLGML)	*Cinnamomum cassia* Presl (Guizhi)<br>*Glycyrrhiza uralensis* Fisch. (Gancao)<br>Longgu (Longgu)<br>*Ostrea gigas* Thunberg (Muli)	Arrhythmia	Fei et al., 2011
Guizhi Decoction (GZ)	*Cinnamomum cassia* Presl (Guizhi)<br>*Paeonia lactiflora* Pall. (Chishao)<br>*Glycyrrhiza uralensis* Fisch. (Gancao)<br>*Ziziphus jujuba* Mill. (Dazao)<br>*Zingiber officinale* Rosc. (Shengjiang)	Arrhythmia	Fei et al., 2011
Guizhi Jia Longgumuli Decoction (GZJLGML)	*Cinnamomum cassia* Presl (Guizhi)<br>*Paeonia lactiflora* Pall. (Chishao)<br>*Zingiber officinale* Rosc. (Shengjiang)<br>*Glycyrrhiza uralensis* Fisch. (Gancao)	Arrhythmia	Fei et al., 2011

	Ziziphus jujuba Mill. (Dazao)		
	Longgu (Longgu)		
	Ostrea gigas Thunberg (Muli)		
Tianma Goutengyin (TMGTY)	*Gastrodia elata* Bl. (Tianma)	Hypertension	Jiao et al., 2017
	Uncaria rhynchophylla (Miq.) (Gouteng)		
	Haliotis diversicolor Reeve (Shijueming)		
	Gardenia jasminoides Ellis (Zhizi)		
	Scutellaria baicalensis Georgi (Huangqin)		
	Cyathula officinalis Kuan (Chuanniuxi)		
	Eucommia ulmoides Oliv. (Duzhong)		
	Leonurus japonicas Houtt. (Yimucao)		
	Taxillus chinensis (DC.) Danser (Sangjisheng)		
	Polygonum multiflorum Thunb. (Shouwuteng)		
	Poria cocos (Schw.) Wolf (zhufushen)		
Xiaojianzhong Decoction (XJZ)	Maltose	Hypertension	Du et al., 1999
	Cinnamomum cassia Presl (Guizhi)		
	Paeonia lactiflora Pall. (Chishao)		
	Glycyrrhiza uralensis Fisch. (Zhigancao)		

	Ziziphus jujuba Mill. (Dazao)		
	Zingiber officinale Rosc. (Shengjiang)		
Xiaochanhu Decoction (XCH)	*Bupleurum chinense* (Chaihu)	Angina pectoris	Du et al., 1999
	Pinellia ternata (Thunb.) (Banxia)		
	Panax ginseng C. A. Mey. (Renshen)		
	Glycyrrhiza uralensis Fisch. (Gancao)		
	Scutellaria baicalensis Georgi (Huangqin)		
	Zingiber officinale Rosc. (Shengjiang)		
	Ziziphus jujuba Mill. (Dazao)		
Wuzhuyu Decoction (WZY)	*Euodia rutaecarpa* (Juss.) Benth. (Wuzhuyu)	Hypertension	Du et al., 1999
	Zingiber officinale Rosc. (Shengjiang)		
	Panax ginseng C. A. Mey. (Renshen)		
	Ziziphus jujuba Mill. (Dazao)		

*: The names of all Chinese medicine are in accordance with the Chinese Pharmacopoeia 2015 edition. The Chinese names of the Chinese medicine are in parentheses.

Supplementary Table 2 CMM-derived small molecule compounds/drugs against cardiovascular diseases

Name	Source	Structural formula	Related targets/ pathways	Function	Experimental mode	Reference
CAG	*Astragalus mongholicus* Bunge (Huangqi)	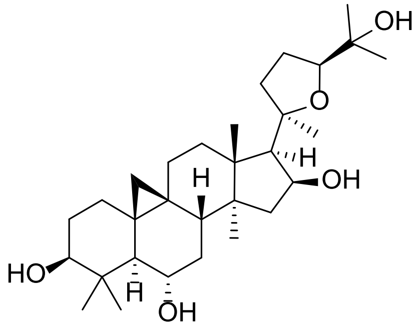	AKT1/ RPS6KB1	Ameliorating left ventricular dysfunction and cardiac remodeling	Heart failure rat model	Wang et al., 2018
CG	*Astragalus mongholicus* Bunge (Huangqi)	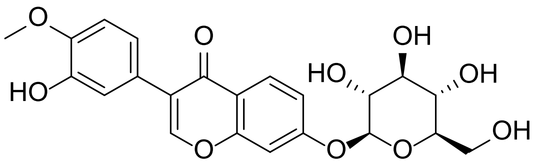	-	Reducing myocardial injury	heatstroke rat model	Tsai et al., 2018
Gastrodin	*Gastrodia elata* Bl. (Tianma)	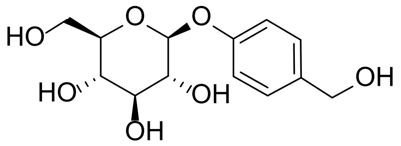	P62/ LC3	Alleviating myocardial ischemia / reperfusion injury	Ischemia - reperfusion mouse model	Fu et al., 2018

Wogonin	*Scutellaria baicalensis* Georgi (Huangqin)	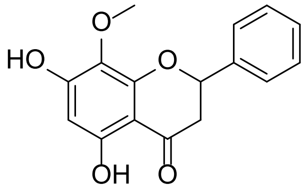	PI3K/ Akt	Attenuating myocardial hypertrophy	Myocardial hypertrophy mouse model; H9C2 cell	Qian et al., 2018
Leonurine	*Leonurus japonicas* Houtt. (Yimucao)	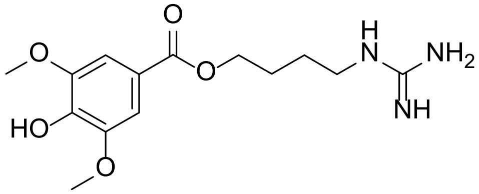	PI3K/ AKT/ GSK3β	Inducing anti-apoptotic effects	Myocardial infarction rat model	Qian et al., 2018
Honokiol	*Magnolia officinalis* Rehd. Et Wils. (Houpu)	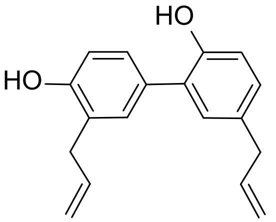	AMPK/ ULK1	protecting against myocardial dysfunction	Rat model of β1-AAB positive with myocardial dysfunction	Wei et al., 2018
APG	*Akebia quinata* (Thunb.) Decne (Mutong)	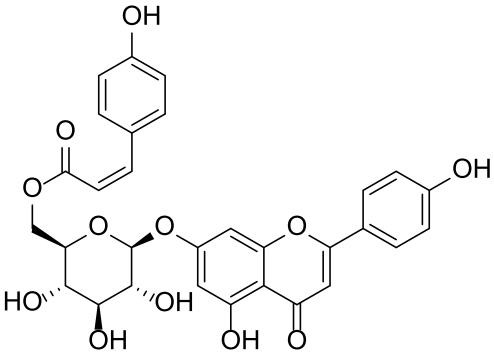	AMPK	Attenuating myocardial ischemia/reperfusion injury	Neonatal rat model	Feng et al., 2018

AMF	*Selaginella tamariscina* (Beauv.) Spring (Juanbai)	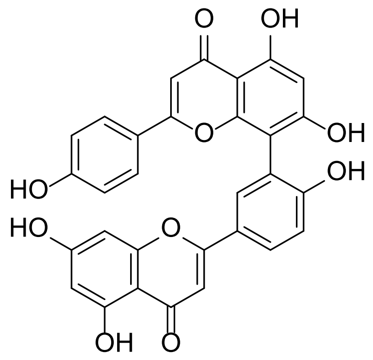	RAS/ NADPH	Inhibiting oxidative stress	Rat model of high fructose and fat diet	Qin et al., 2018
Chicoric acid	*Cichorium glandulosum* Boiss. Et Huet (Juju)	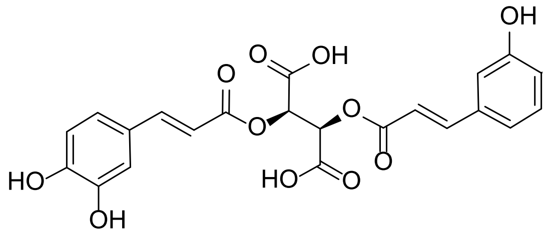	-	Improving heart and blood responses	Hypobaric hypoxia yak model	Wu et al., 2018
Acacetin	*Chrysanthemum morifolium* Ramat. (Juhua)	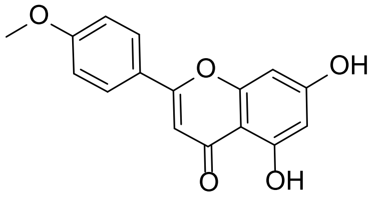	MAPK/ PI3K/ Akt	Inhibiting cardiac hypertrophy and fibrosis	Myocardial infarction mouse model	Chang et al., 2017

Barbaloin	*Aloe barbadensis* Miller (Luhui)	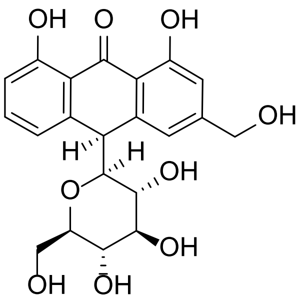	AMPK	Attenuating myocardial ischemia /reperfusion injury	Ischemia - reperfusion rat model	Zhang et al., 2017
Resveratr-ol	*Polygonum cuspidatum* Sieb. Et Zucc. (Huzhang)	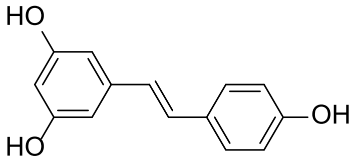	ERK-related pathway	Cardiovascular protective effects	clinical therapeutics	Cho et al., 2017
Quercetin	*Sophora japonica* L. (Huaihua)	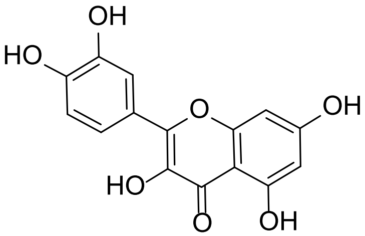	NF-κb pathway	Ameliorating oxidative stress, inflammation and apoptosis	Diabetic rat model	Roslan et al., 2017
Melatonin	*Lycium barbarum* L. (Gouqizi)	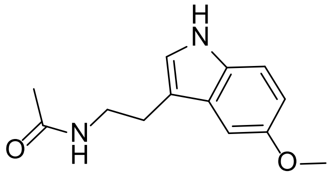	AMPK-PGC-1α-SIRT3 pathway	Ameliorating myocardial ischemia/reperfusion injury	Type 1 diabetic rat model	Yu et al., 2017

Punicalagin	*Punica granatum* L. (Shiliupi)	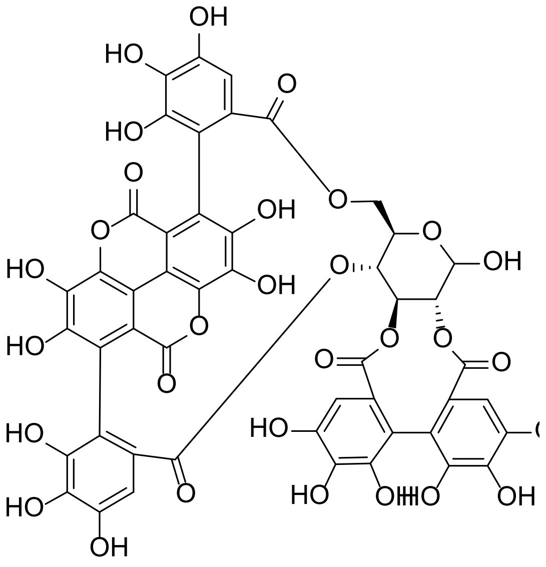	AMPK	Attenuating myocardial ischemia -reperfusion Injury	Ischemia-reperfusion rat model	Ding et al., 2017
Berberine	*Phellodendron chinense* Schneid. (Huangbai)	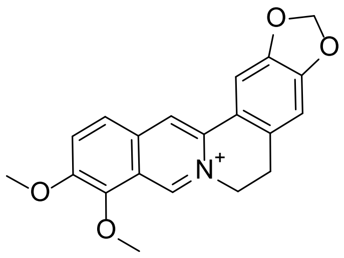	microRNA-29b	Promoting ischemia -induced angiogenesis	Myocardial infarction mouse model	Zhu et al., 2017
Naringenin	*Citrus aurantium* L. (Zhishi)	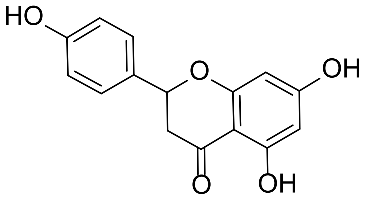	-	Preventing prematurely induced senescence	H9C2 cells senescence Model	Da Pozzo et al., 2017

Diacerein	*Aloe barbadensis* Miller (Luhui)	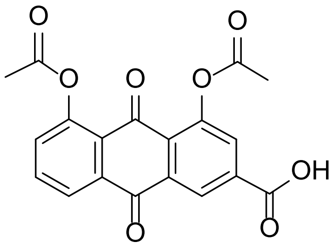	NF-κB	Promoting lower end-systolic and end-diastolic left ventricular volumes	Myocardial infarction rat model	Torina et al., 2015
Luteolin	*Lonicera japonica* Thunb.(Jinyin hua)	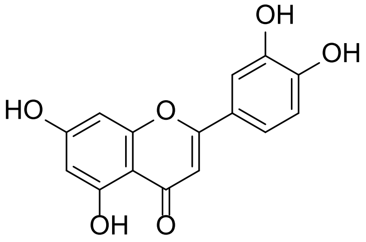	MAPK pathway	Ameliorating the impaired mitochondrial morphology	Ischemia-reperfusion rat model; H9C2 cell	Yu et al., 2015
Piceatann-ol	*Rheum palmatum* L. (Dahuang)	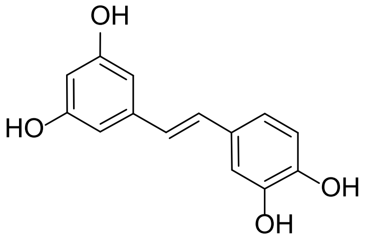	PPAR-α; IL-13; TNF-α; NF-κB; PI3K; HO-1	Prevention of hypercholesterolemia, cardiac arrhythmia, monocyte-endothelial cell adhesion, smooth muscle cell proliferation and migration, endothelial dysfunction,□angiogenesis; Anti-inflammatory effect; Vasorelaxation effect; Antioxidant activity	-	Tang et al., 2014

Trigonelline	Huluba (Huluba)	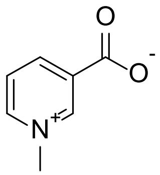	Hsp27 and αB-crystallin	Preventing ISO-induced oxidative stress	Isoproterenol-induced myocardial injury mouse model	Panda et al., 2013
PCA	*Acacia catechu* (L. f.) Willd. (Ercha)	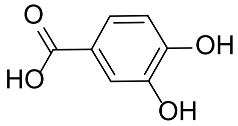	-	Protective effects on TCDD-induced oxidative and histopathological damage	Rat model with TCDD-induced damage	Ciftci et al., 2013
Sal B	*Salvia miltiorrhiza* Bge. (Danshen)	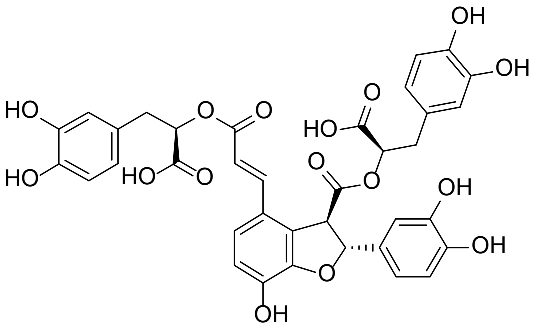	NO production-related pathways	Attenuating myocardial ischemia injury	Myocardial infarction mouse model; Human umbilical vein endothelial cells (HUVECs)	Ciftci et al., 2013
Tan IIA	*Salvia miltiorrhiza* Bge. (Danshen)	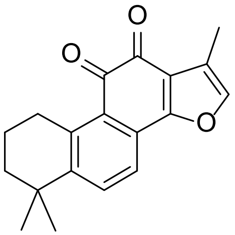	NO production-related pathways	Attenuating myocardial ischemia injury	Myocardial infarction mouse model; Human umbilical vein	Ciftci et al., 2013

					endothelial cells (HUVECs)	
Curcumin	*Curcuma longa* L. (Jianghuang)	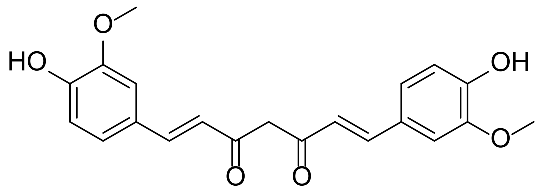	Histone acetyltransferase (HAT)	Beneficial effects on left ventricular systolic function	Myocardial Infarction rat model	Sunagawa et al., 2011
CTS	*Salvia miltiorrhiza* Bge. (Danshen)	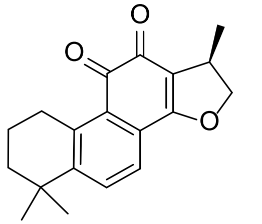	pro-inflammatory cytokines	Inhibiting TNF-α-induced expression of adhesion molecules	Myocardial ischemia/ reperfusion rat model; human umbilical vein endothelial cells (HUVEC)	Jin et al., 2009

AMF: Amentoflavone; APG: Apigenin-7-O-β-D-(-6″-p-coumaroyl)-glucopyranoside; CAG: Cycloastragenol; CG: Calycosin-7-O-β-D-glucoside; CTS: Cryptotanshinone; PCA: Protocatechuic acid; Sal B: Salvianolic acid B; Tan IIA: Tanshinone IIA

Supplementary Figure 1

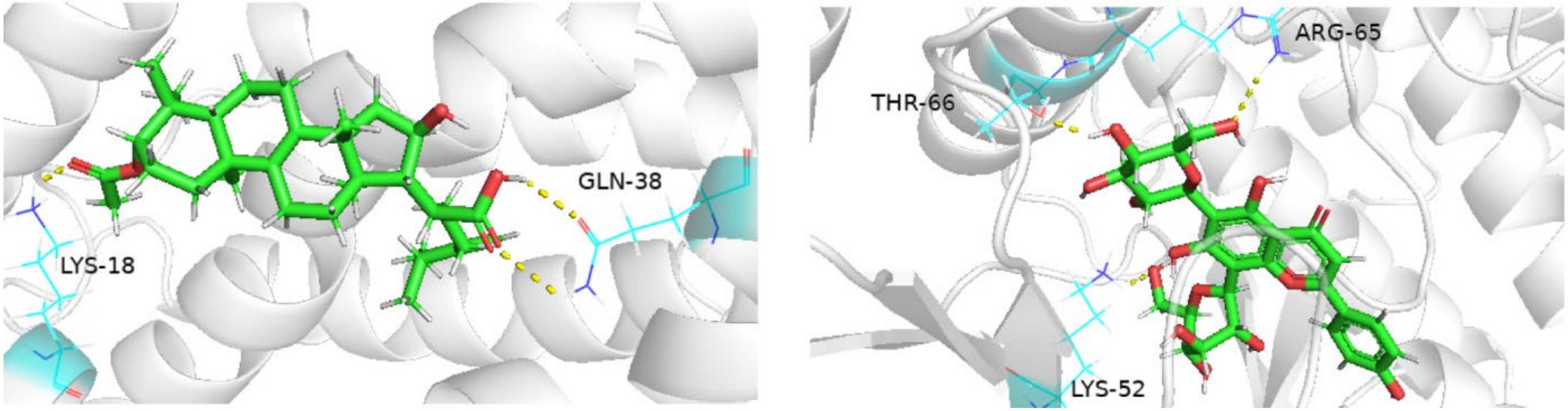

Supplement: Supplementary Table 1 — CCCP against cardiovascular diseases. [file DataSheet_1.pdf]
